# Early warning indicators for severe hemorrhagic fever with renal syndrome

**DOI:** 10.3389/fimmu.2026.1986264

**Published:** 2026-10-26

**Authors:** Xiaojiao Li, Lin Su, Yinli He, Linpei Zhang, Tianyan Chen, Minhong Fei, Yawen Wang

**Affiliations:** 1 Infectious Diseases Department, The First Affiliated Hospital of Xi’an Jiaotong University, Xi’an, Shaanxi, China; 2 BioBank, The First Affiliated Hospital of Xi’an Jiaotong University, Xi’an, Shaanxi, China; 3 Shaanxi Engineering Research Center for Biobank and Advanced Medical Research, The First Affiliated Hospital of Xi’an Jiaotong University, Xi’an, Shaanxi, China; 4 Jiangyan Hospital of Traditional Chinese Medicine, Taizhou, Jiangsu, China; 5 Department of Laboratory Medicine, First Affiliated Hospital, School of Medicine, Xi’an Jiaotong University, Xi’an, Shaanxi, China

**Keywords:** biomarkers, early warning indicators, hemorrhagic fever with renal syndrome, risk stratification, severe HFRS

## Abstract

Hemorrhagic fever with renal syndrome (HFRS) remains a significant public health concern, and early identification of severe cases is critical for improving outcomes. This review synthesizes recent advances in early warning indicators for severe HFRS. Clinically accessible indicators form the basis of early risk stratification. Key bedside signs such as high fever, severe gastrointestinal symptoms, altered mental status, and overt bleeding tendency, together with routine laboratory parameters including thrombocytopenia, leukocytosis, and elevated procalcitonin, have consistently shown robust predictive value for disease severity and mortality. Immunological markers provide mechanistic insight that also supports early identification of severe HFRS, as reflected by a core cytokine imbalance, impaired antigen-specific T- and B-cell responses, and aberrant activation of monocytes, neutrophils, and the complement system. Emerging biomarkers of endothelial barrier disruption, early renal tubular injury, oxidative stress, and metabolic dysregulation may further enhance predictive capacity. Despite these advances, challenges remain regarding external validation and clinical translation. Future research should focus on establishing standardized, multicenter-validated early warning systems to enable precise management of severe HFRS.

## 1 Introduction

Hemorrhagic fever with renal syndrome (HFRS) is an acute infectious disease caused by several *Orthohantavirus* species in the family *Hantaviridae.* Human infection usually occurs through inhalation of aerosolized virus from the excreta of infected rodents (1). The main causative viral strains differ by geographic region. Hantaan virus (HTNV) and Seoul virus (SEOV) are major causes of HFRS in Asia. HTNV infection may be associated with case fatality rates of approximately 5%–15%, whereas SEOV infection is generally milder. Puumala virus (PUUV) predominates in Europe and generally causes a milder form known as nephropathia epidemica (NE), with a case fatality rate of 0.1% to 0.4%. Dobrava virus (DOBV), which circulates mainly in the Balkans and other parts of Europe, has been documented to cause more severe disease (1–3). China accounts for >90% of the global HFRS burden, with more than 1.62 million cases and nearly 47,000 deaths from 1950 to 2014 (4). During 2014–2023, 91,388 total cases were reported, corresponding to an average annual incidence of 0.65/100,000 (5).

Hantaviruses enter host cells primarily by binding to β3 integrins on the surface of endothelial cells (6, 7). Instead of directly lysing endothelial cells, viral infection initiates a strong innate and adaptive immune response, along with an inflammatory cascade. This results in endothelial barrier dysfunction, which can be clinically observed as increased vascular permeability, thrombocytopenia, coagulation disturbances, and acute kidney injury (AKI) (8, 9).

Clinically, HFRS classically manifests five sequential phases: the febrile phase, hypotensive shock phase, oliguric phase, diuretic phase, and convalescent phase. Not all patients experience every phase in complete sequence; phase overlap frequently occurs, in which features of two or more phases appear simultaneously (10). HFRS can be classified into four grades: mild, moderate, severe, and critical. In clinical practice, mild and moderate cases are often grouped together as a broad “mild” category, while severe and critical cases are grouped as “severe” (7, 11–13). However, the criteria for severe disease are not fully consistent across different hantavirus infections. For HTNV infection, severe HFRS is defined by a body temperature >40 °C, neurological symptoms, shock, oliguria lasting ≥5 days or anuria lasting ≥2 days, or hemorrhage in vital organs (10, 14). For PUUV-induced nephropathia epidemica (NE), severe cases are often characterized by severe AKI or shock (15, 16). Despite these differences, the definitions all highlight the essential features of severe HFRS, including hypotension or shock, severe AKI, a significant bleeding tendency, and multiple organ dysfunction (17). Currently, no specific antiviral therapy is available, and the management of severe HFRS relies mainly on continuous renal replacement therapy (CRRT), mechanical ventilation, and vasopressor support (12, 17, 18). However, because of rapid progression to severe disease and the limited therapeutic window, intervention is often delayed, resulting in high mortality rates (14). Therefore, early recognition of patients at risk of severe progression and prompt initiation of intensive care are critical for improving outcomes.

In recent years, research on early warning of severe HFRS has advanced considerably across multiple levels, encompassing bedside signs, routine laboratory parameters, immunological markers, and emerging novel biomarkers. In this review, we comprehensively synthesize progress in the aforementioned areas and evaluate the predictive performance and translational potential of various biomarkers. We also discuss current challenges and future perspectives, with the aim of establishing an evidence base for early identification and precise management of severe HFRS.

## 2 Clinically accessible warning systems for early risk stratification

Clinically accessible warning systems including bedside signs and routine laboratory parameters are the most important indicators for predicting severe HFRS. This section reviews the available evidence on bedside signs and standard laboratory tests for predicting severe HFRS. The goal is to provide a practical framework for early risk assessment.

### 2.1 Bedside signs of severe HFRS prediction

Bedside signs offer unique discriminative value for early risk stratification in HFRS, particularly in the absence of laboratory data. The 2021 Chinese Expert Consensus on Prevention and Treatment of HFRS identifies several key warning indicators for severe cases. These include a history of travel to endemic areas or contact with rodents and their excreta within two months prior to onset, high fever (temperature >40 °C or febrile duration exceeding one week), severe gastrointestinal symptoms such as intense nausea and vomiting, psychiatric disturbances including agitation or delirium, marked conjunctival edema, and obvious bleeding tendency (10). Regarding imaging findings, a retrospective analysis by Huang et al. of 191 patients with HFRS in southwestern China demonstrated that pleural effusion was an independent risk factor for progression to severe HFRS. Patients with pleural effusion had a 4.49-fold higher risk of severe progression compared to those without it, suggesting that this sign may reflect more extensive capillary leakage and systemic inflammation (14).

The quick Sequential Organ Failure Assessment (qSOFA) score also has shown significant value in predicting severe HFRS. Ma and colleagues conducted a multicenter study of 551 HFRS patients in Hubei Province. They demonstrated that an admission qSOFA score of 2 or higher was an independent risk factor for 30-day mortality. The qSOFA score is defined as meeting any two of the following criteria: respiratory rate ≥22 breaths/min, systolic blood pressure ≤100 mmHg, or altered mental status. This finding suggests that the qSOFA score, without reliance on any laboratory tests, can serve as a rapid triage tool for early identification of high-risk HFRS patients and for determining the need for intensive care unit admission (19).

Notably, the early warning indicators for severe HFRS vary across different age groups. Researchers at Tangdu Hospital have conducted stratified warning analyses for both older adults and children (20, 21). In the older-adult cohort (≥ 60 years), Li et al. reviewed 146 cases and identified several early warning signs using multivariate logistic regression. These included chest tightness or dyspnea, nausea or vomiting, decreased urine output (UO), skin and mucosal hemorrhage, and phase overlap (disease course spanning two or more typical phases). These findings suggest that assessment of older adults with HFRS should focus more on cardiopulmonary function, gastrointestinal symptoms, renal perfusion, coagulation status, and disease progression patterns (20). In the pediatric cohort (<16 years), the same group analyzed 103 cases. They found that conjunctival edema, hypotensive shock, and elevated serum creatinine (Cr) were key early warning indicators. This suggests that assessment in children should focus more on fluid extravasation, circulatory status, and early renal changes. Taken together, risk assessment should combine exposure history, consensus-based warning signs, bedside scores, and age-specific findings (21).

### 2.2 Routine laboratory parameters for early warning of severe HFRS

#### 2.2.1 Blood cell indices and inflammatory markers

Blood count and routine inflammatory markers are the most accessible laboratory indicators for evaluating the systemic host response in HFRS. Markedly elevated white blood cell count (WBC) and severely reduced platelet count (PLT) have long been recognized as warning signs for HFRS severity (11, 22). The 2021 Expert Consensus has established WBC >30 × 10^9^/L and PLT <20 × 10^9^/L as definitive severity warning thresholds (10). This pattern has been consistently confirmed by multiple retrospective cohort studies from various regions. Yang et al. analyzed 109 HFRS cases in central China and found that the median WBC in the severe group (25.2 × 10^9^/L) was significantly higher than in the mild group (12.6 × 10^9^/L), while the median PLT (22 × 10^9^/L) was significantly lower than in the mild group (54 × 10^9^/L) (12). Chen et al. further examined the association between these two indicators and adverse outcomes in a cohort of 395 HFRS patients in northwestern China. The results showed that the median WBC in the non-survivor group (23.19 × 10^9^/L) was significantly higher than in the survivor group (13.29 × 10^9^/L), while the median PLT (22 × 10^9^/L) was significantly lower than in the survivor group (41 × 10^9^/L) (23). Platelet distribution width (PDW) may provide information on platelet activation and function beyond that conveyed by PLT alone. Fan et al. studied 260 HFRS patients and found that PDW on the first day of admission increased significantly with disease severity. Of note, in critically ill patients, the association between PDW and prognosis was reversed, with non-survivors exhibiting lower PDW than survivors. This reversal may reflect exhausted marrow hematopoietic compensation, where declining PDW portends worse outcomes (24).

The neutrophil-to-red blood cell ratio (NRR) and neutrophil-to-lymphocyte ratio (NLR), as simple readouts of systemic inflammation, have demonstrated predictive value across multiple viral species. Xiao et al. analyzed 342 HTNV-HFRS cases in Xi’an and found that NRR was significantly elevated in the severe/critical group, with optimal discriminative performance for severity grading, whereas NLR was significantly elevated in the non-survivor group, with optimal performance for 28-day all-cause mortality prediction. Both markers are accessible at the bedside, with NRR more suitable for early severity stratification and NLR better aligned with mortality risk assessment (25). In PUUV infection, Nusshag et al. analyzed 32 PUUV-infected patients in Germany and showed that peak NLR was significantly elevated in severe patients with hospitalization exceeding five days, suggesting that systemic neutrophil-lymphocyte imbalance is an important accompanying feature of PUUV severity (26).

Procalcitonin (PCT), as an indicator reflecting the severity of infection and inflammation, has shown predictive value across multiple independent studies. Duan et al. found that serum PCT level at emergency department admission was an independent risk factor for severe progression in HFRS patients, with the median PCT in the severe group significantly higher than in the mild group (8.12 vs. 1.39 ng/mL); an admission PCT exceeding 2.97 ng/mL demonstrated good predictive value for severity, with an area under the receiver operating characteristic curve (AUC) of 0.862, and a sensitivity of 88.0% (27). Chen et al. found in 272 HFRS patients that admission PCT levels increased significantly with disease severity, with an AUC of 0.792 for predicting 28-day survival/death at an optimal cutoff of 2.28 ng/mL, outperforming WBC (AUC 0.769) (28). Che et al. further confirmed in a retrospective study of 373 HFRS patients that the median admission PCT in the severe group was significantly higher than in the mild group (3.8 vs. 1.3 ng/mL), with an AUC of 0.824 for predicting severity; PCT was also significantly higher in non-survivors than in survivors (3.6 vs. 1.7 ng/mL), with an AUC of 0.737 for predicting hospital mortality (7). Huang et al. confirmed in a retrospective cohort of 191 HFRS patients in southwestern China that PCT was an independent risk factor for severe progression, with each 1 ng/mL increase in PCT associated with approximately 33% higher odds of developing severe disease (14). Yu et al. also confirmed in a cohort of 384 HFRS patients that PCT was significantly higher in the severe/critical group than in the mild/moderate group, with multivariate logistic regression confirming elevated PCT as an independent predictor of severe progression (29). These results indicate that PCT is a useful marker for assessing both disease severity and short-term mortality in HFRS, and may help identify high-risk patients early.

Unlike PCT, C-reactive protein (CRP) showed limited independent value for HFRS severity prediction. Che et al. studied 373 HFRS patients and found that admission CRP was significantly higher in the severe group than in the mild group (median 42.1 vs. 29.5 mg/L). However, its AUC for severity prediction was only 0.614, far below that of PCT (0.824) (7). Yang et al. also found that although the median CRP was significantly higher in the severe group than in the mild group (51.0 vs. 30.9 mg/L), CRP was deemed to lack independent incremental value during least absolute shrinkage and selection operator (LASSO) regression variable selection and was therefore not included in the final prediction model (12). Chen et al. further confirmed in 272 HFRS patients that admission CRP was elevated across all severity grades (mean approximately 30.1 to 33.96 mg/L for each group), with no statistically significant difference among the four groups, indicating that CRP does not exhibit a gradient change with disease severity. However, CRP retains potential utility in assessing secondary bacterial infection: the median CRP in the infected group was significantly higher than in the non-infected group (43.2 vs. 26.4 mg/L, p < 0.001), with an AUC of 0.588 for predicting secondary bacterial infection (p = 0.005). Although this represents moderate-to-weak performance, it is statistically significant, whereas PCT showed no discriminative capacity in this setting (28). Therefore, CRP is more suitable as an auxiliary marker for bacterial infection rather than an independent predictor of HFRS severity.

The predictive role of serum albumin (ALB) and hemoglobin (Hb) remains controversial. The 2021 Expert Consensus lists serum ALB below 15 g/L as a severity warning indicator (10). Huang et al. reported that the median admission ALB was significantly lower in the severe group than in the mild group (25.45 vs. 29.80 g/L) on univariate analysis. After LASSO screening, multivariate logistic regression confirmed ALB as an independent protective factor against severe progression. Each 1 g/L increase in ALB was associated with an approximate 14% reduction in HFRS severity risk (14). However, both Duan et al. and Yang et al. demonstrated that although ALB was lower in the severe group at the univariate level, it was not an independent risk factor in multivariate models, suggesting that its predictive power might be affected by other coexisting indicators (12, 27). Hb findings are also inconsistent. Huang et al. found no significant difference in admission Hb between severe and mild groups (14), whereas Yang et al. reported significantly lower Hb in severe cases, though this association did not persist in multivariate analysis (12). These findings indicate that the independent value of ALB and Hb as severity warning indicators is not yet stable and should be interpreted in conjunction with other indicators.

#### 2.2.2 Coagulation function assessment

Coagulation and fibrinolysis system disorders represent core processes in HFRS severity progression and are major causes of severe hemorrhage and death. Multiple studies have confirmed that severe HFRS patients present with typical coagulation activation and consumption at admission. In a cohort of 395 patients with HTNV-HFRS, Chen et al. found that non-survivors exhibited significantly prolonged prothrombin time (PT) and activated partial thromboplastin time (APTT), sharply decreased fibrinogen (Fib), and markedly elevated D-dimer and fibrinogen degradation products (FDP) at admission. The study also found that the abnormal detection rates of D-dimer and FDP were significantly higher than those of PT, APTT, and Fib, suggesting that early hyperfibrinolysis dominates the coagulation disorders in HFRS and should be a priority in routine assessment (23).

Disseminated intravascular coagulation (DIC) represents the end-stage of coagulation failure. Sundberg et al. applied the International Society on Thrombosis and Hemostasis (ISTH) criteria to perform DIC scoring in 106 patients with HFRS and found that approximately 18.9% to 28.3% of the patients met the diagnostic criteria for overt DIC. DIC status was significantly associated with moderate to severe disease and bleeding. Notably, a modified ISTH scoring template incorporating the fibrinogen-to-CRP ratio to adjust for inflammatory influence demonstrated superior predictive performance. These findings suggest that DIC scoring could serve as an effective risk stratification tool in HFRS (30).

Thrombomodulin (TM) has been recognized as a marker of endothelial injury in recent years. Studies on HTNV-induced HFRS have demonstrated that plasma soluble thrombomodulin (sTM) levels increase in parallel with disease severity, with the highest levels observed in critically ill patients. In this context, sTM achieved an AUC of 0.872 for predicting disease severity (31). Another independent study further confirmed that sTM levels are closely associated with the degree of renal impairment, and its predictive performance for 28-day mortality significantly outperforms traditional coagulation parameters such as PT and APTT (32). This marker thus supplements the endothelial injury dimension not captured by routine coagulation indicators.

Thromboelastography (TEG) can objectively reflect the dynamics of HFRS coagulation. Dou et al. conducted a retrospective study of 342 HFRS patients (predominantly HTNV) in Nanchang and found that HTNV-infected patients presented with hypocoagulable features within 24 hours of admission, with more pronounced changes in the critically ill group, manifested as prolonged reaction time (R) and clot-formation time (K), together with decreased alpha angle, maximum amplitude (MA), and coagulation index (CI), reflecting progressive inhibition of coagulation factor and platelet function (33). Overall, coagulation assessment in HFRS should cover multiple dimensions, including routine parameters, fibrinolysis markers, DIC scores, endothelial injury indicators, and dynamic monitoring. Early elevations in D-dimer and FDP, along with sTM levels, deserve particular attention.

#### 2.2.3 Renal, urine, and electrolyte examination

AKI is one of the most common organ injuries in HFRS, but the predictive value of routine renal function indicators requires nuanced interpretation. Chen et al. found in a cohort of 395 HFRS patients that although the incidence of AKI during the acute phase was as high as 50.76%, Cr levels did not differ significantly between survivors and non-survivors after standardized supportive therapy including blood purification. This finding suggests that under active intervention, the degree of renal impairment alone may have limited independent predictive value for mortality (23).

UO, as an earlier indicator of renal function changes, may have greater predictive value. Lee et al. studied 26 HTNV-infected HFRS patients and found that although mean UO did not differ significantly between severe and mild/moderate groups, low UO itself was significantly associated with a severe immune phenotype, and its decrease correlated with elevated levels of certain inflammatory factors (such as CXCL10) in serum (34). This indicates that UO is not just a standard indicator of glomerular filtration but also a more sensitive measure for detecting severe conversion signals associated with systemic immunoinflammatory dysregulation.

Compared to UO as a macroscopic indicator, analysis of urine composition provides more direct warning information from the renal parenchymal injury level. Mantula et al. studied 205 PUUV-infected patients in Finland and showed that a urine dipstick albumin of 2+ or higher at admission could early predict 89% of subsequent severe AKI. The peak of glomerular proteinuria components (albumin/IgG) preceded the Cr peak by four days, providing a warning window earlier than Cr elevation (35). Nusshag et al. analyzed 22 PUUV-HFRS patients and showed that the degree of glomerular injury (urinary nephrin, urinary IgG) was significantly correlated with hospitalization duration, peak serum Cr, peak proteinuria, and hypoalbuminemia. This indicates that glomerular barrier disruption is a critical driver of clinical severity in PUUV infection (36).

Regarding electrolytes, Rista et al. prospectively analyzed 33 DOBV-HFRS patients in Albania and found that admission serum sodium ≤133 mmol/L predicted severe AKI with an AUC of 0.957, outperforming PLT and WBC counts (37). Additionally, indicators reflecting cardiorenal interaction deserve attention. Plasma B-type natriuretic peptide (BNP) is a classical circulating marker reflecting ventricular wall tension and volume load. In renal insufficiency, it accumulates due to decreased renal clearance, making it a sensitive circulating indicator for assessing cardiorenal interaction (38). Zhao et al. studied 176 HTNV-HFRS patients and 41 healthy controls and found that BNP levels were significantly elevated during the acute phase of HFRS and increased with disease severity. BNP levels were positively correlated with Cr, urea, and APTT, and negatively correlated with PLT and Fib, indicating that its fluctuation is closely associated with HFRS severity and that it is a candidate plasma marker for predicting severe disease (39). In summary, assessing renal function should not be limited to Cr monitoring but should also consider dynamic changes in UO, urinary protein makeup, electrolytes, and cardiorenal interaction markers for earlier and more thorough evaluations.

#### 2.2.4 Liver function indicators

Liver dysfunction is not uncommon in HFRS patients and often occurs as part of multiple organ dysfunction syndrome (MODS) (7). Multiple independent studies have consistently demonstrated that elevated total bilirubin (TBIL) at admission is an important warning signal for HFRS severity and mortality. Chen et al. found that the median admission TBIL was significantly higher in non-survivors than in survivors, with hyperbilirubinemia (>17.1 μmol/L) present in 48.00% of the non-survivor group versus 23.50% of the survivor group. Multivariate logistic regression analysis confirmed that elevated TBIL was an independent risk factor for mortality (23). Yu et al. analyzed 384 HFRS patients in Zhejiang and showed that the mean TBIL was significantly higher in the severe group than in the mild/moderate group, with multivariate logistic regression confirming TBIL elevation as an independent predictor of severe progression (29). Che et al. provided complementary evidence from the perspective of liver injury incidence in 373 HFRS patients. The incidence of liver injury in non-survivors was 100%, significantly higher than the 68.2% in survivors. Further analysis of the liver enzyme profile revealed that non-survivors not only had significantly elevated alanine aminotransferase (ALT), but also showed synchronous and marked increases in lactate dehydrogenase (LDH) and alpha-hydroxybutyrate dehydrogenase (alpha-HBDH) (7). These changes reflect hepatocyte membrane integrity disruption and metabolic dysfunction, suggesting more extensive and severe hepatic parenchymal injury in non-survivors.

Aspartate aminotransferase (AST) elevation is another noteworthy warning indicator. Ma et al. identified AST ≥161 IU/L as an independent predictor of 30-day mortality when constructing the EASTAR score. The study emphasized that in HFRS, early AST elevation is primarily related to skeletal and cardiac muscle injury secondary to capillary damage rather than being an exclusive indicator of hepatic failure alone, and should be viewed as a cross-tissue nonspecific injury signal (19). He et al. further found in a retrospective analysis of 317 HFRS patients in Xi’an that the AST-to-platelet ratio index (APRI) was an independent predictor of mortality, with an AUC of 0.95 for predicting death, outperforming age and NLR; patients with APRI ≥6.15 had significantly lower survival rates, suggesting that APRI as a composite indicator integrates dual information on hepatic injury and platelet consumption and possesses high predictive efficacy (40).

Serum ferritin is another indicator with important predictive value. Che et al. found in a retrospective study of 373 HFRS patients that serum ferritin levels increased significantly with disease severity and mortality risk, with an AUC of 0.732 for predicting severity (optimal cutoff approximately 3,550 ng/mL) and an AUC of 0.853 for predicting 30-day mortality (optimal cutoff approximately 10,700 ng/mL), significantly outperforming PCT (AUC 0.737 for mortality) and CRP (0.703) in the same cohort. This finding suggests that ferritin may serve as a strongly associated marker and potential warning indicator for HFRS severity and mortality (7). Collectively, liver function and related indicators have important value in HFRS severity prediction, with TBIL as an independent risk factor, APRI as a composite indicator, and ferritin as a strongly associated marker warranting particular attention.

### 2.3 Prediction models based on bedside signs and routine laboratory parameters

The preceding sections have reviewed the evidence for individual routine indicators in HFRS severity prediction. However, the pathophysiology of HFRS involves interactions among inflammation, coagulation, endothelial injury, renal perfusion, and multi-organ damage. Single indicators often cannot fully reflect this complex network, and their sensitivity or specificity is often limited. Therefore, integrating indicators that reflect different pathological processes to construct combined prediction models has become a major trend in improving predictive efficacy (**Supplementary Table S1**).

The early exploration of combined warning models primarily employed simple combinations of a few routine indicators. In 2007, Kim et al. retrospectively analyzed 61 HTNV-infected patients in South Korea and found that WBC ≥14 × 10^9^/L, AST ≥110 U/L, and microscopic hematuria ≥5/high-power field (HPF) at admission were independent predictors of subsequent oliguric acute renal failure (ARF) (41). In 2014, Du et al. retrospectively analyzed 356 HFRS patients at Tangdu Hospital in Xi’an and constructed a mortality prognostic risk model using multivariate logistic regression, which incorporated four core indicators: WBC, AST, PT, and Fib (42). Although relatively simple, these four indicators covered key pathological dimensions including inflammation, hepatic injury, and coagulation disorders, laying the foundation for subsequent combination frameworks. In 2015, Latus and colleagues constructed a simple scoring system based on a prospective study of 456 PUUV-associated NE patients, incorporating three bedside indicators: thrombocytopenia (PLT <150 × 10^9^/L), elevated CRP (>10 mg/L), and proteinuria (≥1+). It achieved an AUC of 0.71 for predicting subsequent severe AKI in patients with normal admission renal function (43). In a cohort of 164 HFRS patients, Duan et al. achieved a combined detection AUC of 0.900 with just three conventional laboratory parameters, namely WBC, PLT, PCT. This finding further indicates that even the most basic laboratory indicator combinations can achieve excellent predictive performance (27). Wang et al. studied 570 HFRS patients at Xi’an Eighth Hospital with critical illness as the endpoint. Multivariate logistic regression identified hypotensive shock, elevated myoglobin, and elevated neutrophil count as independent predictors. Based on these factors, they constructed a nomogram that achieved AUCs of 0.889 and 0.911 in the training and validation cohorts, respectively, indicating robust predictive performance (18).

Subsequent model construction strategies became increasingly diversified. In 2017, Yu et al. identified decreased PLT, elevated PCT and TBIL, and positive fecal occult blood as four independent predictors of HFRS, with a combined AUC of 0.890. Based on these findings, they constructed the hemorrhagic fever-specific Sequential Organ Failure Assessment (H-SOFA) score, which innovatively incorporated fecal occult blood grading into the traditional Sequential Organ Failure Assessment (SOFA) framework, further improving discriminative performance to 0.98 (29). In 2021, Yang and colleagues conducted a retrospective analysis of 155 HFRS patients and identified six key indicators: neutrophils, Hb, PLT, Cr, calcium, and dyspnea. These variables were then used to construct a nomogram for HFRS severity classification, yielding excellent predictive performance with AUCs of 0.969 and 0.934 in the training and validation sets, respectively (12). These findings demonstrated that routine blood count, renal function, and electrolyte tests, and symptom assessment may support rapid bedside identification of high-risk patients. In 2022, Xie et al. retrospectively analyzed 101 HFRS patients in Changsha and identified WBC, ALB, PT, and creatine kinase-MB (CK-MB) as four independent predictors, with a combined AUC of 0.950 for severity prediction (44). In 2023, Chen et al. stratified 235 HFRS patients into two groups according to the presence of fever at admission and constructed separate predictive models for each. The febrile group model used glomerular filtration rate (GFR), peak high-power field urinary WBC, hyponatremia, elevated urine conductivity, and urinary epithelial shedding as warning indicators, while the afebrile group model employed GFR, hyponatremia, elevated urine conductivity, and elevated cystatin C (CysC). The internal and external validation AUCs for the febrile group model were 0.92 and 0.96, respectively, and the afebrile group model yielded corresponding values of 0.93 and 0.98, precisely matching the pathophysiological characteristics of different presentation time windows (45).

In recent years, model construction has further emphasized multicenter integration and comprehensive consideration of clinical phenotypes. Novel factors reflecting organ crosstalk, such as the cardiorenal interaction and the thyroid-metabolic axis, have been added. In 2025, Ma et al. constructed the EASTAR score based on 551 patients from multicenter data in Hubei, incorporating red blood cell count (≥4.87 × 10¹²/L), age (≥61 years), qSOFA score (≥2), disease phase overlap, and AST (≥161 IU/L) into the model. Taking 30-day mortality as the endpoint, the model achieved an AUC of 0.910 in the validation cohort. Thus, by combining bedside assessment available at admission with demographic characteristics and a single enzymatic marker, this model provides a practical tool for rapid triage (19). Dou et al. studied 342 HTNV-infected patients in Nanchang and developed an early warning model combining TEG parameters within 24 hours of admission with blood count and biochemical indicators. The model achieved an AUC of 0.906 in the training set and 0.762 in external validation, providing the first evidence for the incremental value of TEG dynamic monitoring in early identification of critical HFRS (33). In the same year, Huang et al. identified PCT elevation, ALB decrease, decreased platelet-to-albumin ratio (PAR), and pleural effusion as four independent predictors of severity in a retrospective cohort of 191 HFRS patients in Dali, Yunnan, with a nomogram AUC of 0.890 (14). The same team conducted a retrospective study of 373 HFRS patients in the Dali region and identified prolonged PT, elevated WBC, elevated LDH-to-albumin ratio (LAR), and decreased free triiodothyronine (FT3) as independent predictors of 30-day mortality. Based on these variables, a nomogram prediction model was constructed with an AUC of 0.946. This study was the first to establish the potential value of FT3 and LAR as early warning indicators for mortality in HFRS (46). Concurrently, in a 176 HTNV-HFRS cohort in Xi’an, Zhao et al. found that the combination of plasma BNP, PLT, urea, and Cr achieved an AUC of 0.921 for predicting severe disease, highlighting the monitoring value of cardiorenal interaction indicators (39).

Overall, these models reveal several patterns: first, nearly all successful models include PLT, WBC or PCT as core indicators reflecting coagulation and inflammation, suggesting they are key drivers of HFRS severity. Second, the incorporation of clinical phenotypes such as age, qSOFA score, pleural effusion, and phase overlap can significantly enhance model predictive capacity, particularly when complex test results are not rapidly available, where the value of bedside assessment is especially prominent. Furthermore, the emergence of novel composite indicators such as PAR, LAR, and FT3 provides new directions for optimizing model precision.

## 3 Immune signatures associated with severe HFRS

Despite the wide availability of routine clinical parameters, their specificity is often limited, and marked abnormalities often become evident only after organ injury has already occurred. Immune responses, ranging from cytokine storms to immune cell remodeling, play a central role in the pathogenesis and progression of HFRS. Therefore, systematic evaluation of the predictive value of immunological markers is essential for enhancing our understanding of early detection of high-risk patients. This section comprehensively reviews the roles of cytokines and chemokines, T cells, B cells, the monocyte-macrophage system, neutrophils, and other immune effector molecules in HFRS severity prediction.

### 3.1 Cytokine and chemokine profiles linked to HFRS severity

The cytokine network plays a central role in HFRS immunopathology. Numerous studies have shown that the levels of cytokines and chemokines during the early phase of the disease are closely associated with the severity of HFRS (9, 47, 48). Among proinflammatory cytokines, the predictive value of interleukin-6 (IL-6) and tumor necrosis factor alpha (TNF-α) has been consistently validated across multicenter and multi-cohort studies. Outinen et al. performed a prospective cohort study of 118 patients with PUUV-associated HFRS. They demonstrated that patients with plasma IL-6 levels above the median value of 14.05 pg/mL had a significantly increased risk of elevated Cr, thrombocytopenia, and hospitalization lasting more than seven days (49). Libraty et al. from the same center further confirmed that the maximum plasma IL-6, urinary IL-6, and urinary interleukin-8 (IL-8) concentrations during the acute phase were positively correlated with the severity of AKI (16). This pattern has been consistently replicated across Chinese HTNV-infected cohorts. You et al. conducted a stratified analysis of 37 HFRS patients in Liaoning and demonstrated that serum levels of IL-6, TNF-α, and interleukin-1 (IL-1) increased progressively with the clinical classification from mild to moderate, severe, and critical (50). Yang et al. demonstrated that IL-6 was an independent risk factor for progression to severe/critical disease in a cohort of 118 HFRS patients from Xi’an, with an optimal cutoff of 42.67 pg/mL (11). Wang et al. also studied 35 patients with HTNV-HFRS in Xi’an and observed that the levels of IL-6, TNF-α, and IL-8 increased progressively with clinical severity classification (51). Similarly, Guo et al. reported that higher serum concentrations of TNF-α, IL-6, and interleukin-10 (IL-10) were significantly and positively correlated with the clinical severity of HFRS (52).

It should be noted that cytokines may exert divergent roles in HFRS pathogenesis. Khaiboullina et al. reported that elevated serum levels of interferon gamma (IFN-γ) and interleukin-12 (IL-12) in hantavirus-infected patients were associated with milder clinical manifestations. Patients with high IFN-γ levels experienced shorter febrile and oliguric phases and less hemorrhage, suggesting that T helper 1 (Th1)-type immune responses may confer a protective effect against HFRS severity (53). Reduced levels of the anti-inflammatory cytokine transforming growth factor beta (TGF-β) were also significantly associated with severe disease. A retrospective study analyzing serum samples from 77 patients with acute PUUV-associated NE found that the severe group had significantly lower serum TGF-β1 and TGF-β2 concentrations than the mild group, and that TGF-β levels were positively correlated with PLT count and negatively correlated with Cr (54). Additionally, Zhang et al. found that plasma interleukin-15 (IL-15) during the acute phase of HTNV infection increased with disease severity, with significantly higher levels in severe cases than in mild cases. IL-15 was positively correlated with elevated Cr, reduced PLT, and viral load, suggesting that IL-15 may also serve as a potential indicator of disease severity (55).

Regarding chemokines, monocyte chemoattractant protein-1 (MCP-1), also known as C-C motif chemokine ligand 2 (CCL2), and CXCL10, also known as interferon gamma-induced protein 10 (IP-10), both exhibit substantial predictive potential for disease severity. Korva et al. analyzed 100 patients with HFRS in the Balkan region and found that MCP-1 levels were significantly higher in the severe group of patients infected with DOBV than in those with mild disease (56). Wang et al. further confirmed that acute phase MCP-1 concentrations were significantly elevated in patients with severe or critical disease relative to those with mild or moderate disease, in an HTNV infected cohort from Xi’an. MCP-1 was positively correlated with WBC, blood urea nitrogen (BUN), and Cr, and showed a strong negative correlation with PLT (57). With regard to CXCL10, Korva et al. found that CXCL10 elevation was more pronounced in the severe group of patients infected with PUUV (56). Zhang et al. demonstrated that serum CXCL10 levels were positively correlated with BUN, Cr, WBC, and viral load, and negatively correlated with PLT (58). Also, a Korean study of HTNV-HFRS reported that CXCL10 levels were higher in severe patients than in those with mild or moderate disease and healthy controls (34).

Collectively, early detection of cytokines and chemokines may provide an important immunological basis for the early warning of severe HFRS. At the cytokine level, elevated proinflammatory mediators such as IL-6 and TNF-α, along with reduced levels of protective cytokines such as IFN-γ and TGF-β, together define the core features of disease progression toward severity. At the chemokine level, aberrant elevation of MCP-1 and CXCL10 reflects excessive monocyte-macrophage chemotaxis and T cell recruitment.

### 3.2 Predictive potential of immune signatures in severe HFRS

#### 3.2.1 T-cell responses and subset remodeling

The proportions and phenotypic changes of peripheral blood lymphocyte subsets represent an important window into HFRS severity. Accumulating evidence indicates that the strength of antigen-specific T cell responses is a key determinant of HFRS outcome, and multiple T cell subsets have shown good discriminatory capacity for HFRS severity.

An attenuated antigen-specific T cell response represents an immunological defect that favors progression to severe HFRS. Tang et al. conducted a prospective cohort study of 76 HTNV-HFRS patients in Xi’an. Using human leukocyte antigen E (HLA-E)*01:03-restricted HTNV nonapeptides, they identified six HLA-E-restricted epitope-specific CD8^+^ T cell populations. In patients with mild or moderate disease, the frequency of these cells, the proportion of CD38^+^HLA^-^DR^+^ activated cells, the secretion of IFN-γ, interleukin-2 (IL-2), and granzyme B, and the Ki-67 proliferation rate were all significantly higher than in patients with severe or critical disease. These findings suggest that an inadequate HLA-E-restricted HTNV-specific CD8^+^ T cell response may impair antiviral clearance (59). Studies using enzyme-linked immunospot (ELISPOT) assays to detect HTNV nucleoprotein-specific IFN-γ secretion have further supported this notion. At the earliest accessible time point, 5 to 8 days after fever onset, the total frequency of HTNV-specific IFN-γ-secreting T cells was significantly higher in patients with mild or moderate HFRS than in those with severe or critical disease. This frequency was also higher in patients who subsequently developed only mild renal injury, indicating that a robust HTNV-specific IFN-γ response is a protective factor against severe renal failure (60). Ma et al. examined a cohort of 95 HTNV-HFRS patients in Xi’an and found that HTNV envelope glycoprotein Gn/Gc-specific CD4^+^ T cell responses were negatively correlated with plasma HTNV RNA load. Patients with mild or moderate disease exhibited broader epitope recognition and stronger CD4^+^ T cell responses against Gn/Gc than those with severe disease. These results suggest that a weak Gn/Gc-specific CD4^+^ T cell response may serve as an immune defect associated with insufficient antiviral clearance (61). In a separate study by the same team involving 37 patients with HTNV-HFRS, the frequencies of HLA-A2- and HLA-B35-restricted HTNV nucleoprotein epitope-specific CD8^+^ T cells during the acute phase were higher in mild/moderate than in severe/critical patients. Moreover, these frequencies were negatively correlated with peak Cr and positively correlated with the lowest PLT, further supporting the role of antigen-specific CD8^+^ T cell responses in attenuating renal injury and disease severity (62).

In addition to antigen-specific responses, the remodeling of T cell subsets also holds predictive value for HFRS severity. Longitudinal studies of HTNV-HFRS showed that during the febrile phase, the proportion of CD8^+^ effector memory T (Tem) cells was significantly higher in severe than in moderate patients, whereas during the oliguric phase, the proportion of CD4^+^ central memory T (Tcm) cells was lower in severe than in moderate patients. These observations indicate that dynamic changes in T cell subsets can reflect disease progression trends (63). Among emerging T cell subsets, Zhao et al. systematically analyzed peripheral blood T cell phenotypes in 213 HFRS patients and found that the frequency of CD4^+^CD8^+^ double-positive (DP) T cells increased significantly with disease severity, positively correlated with Cr, uric acid (UA), and urea, and negatively correlated with PLT. Receiver operating characteristic (ROC) analysis showed that this subset predicted severe/critical disease with an AUC of 0.956, significantly outperforming PLT or Cr, suggesting that DP T cells are a sensitive lymphoid marker for HFRS severity stratification (64). In a cohort of 68 patients with HTNV-HFRS in Xi’an, Liu et al. found that the proportion of CD8^low^CD100^−^ cells was negatively correlated with plasma HTNV load. Moreover, this subset was significantly higher in HTNV-specific CD8^+^ T cells in patients with mild or moderate disease than in those with severe or critical disease, indicating that inadequate early expansion of this subset is a phenotypic marker of impaired antiviral clearance (65).

Regulatory T cells (Treg) play a key role in suppressing excessive immune responses, but their overactivation may exacerbate disease by inhibiting effector T cell-mediated antiviral clearance. Koivula et al. performed a flow cytometric analysis of 24 patients during the acute phase of PUUV infection. They found that the mean fluorescence intensity (MFI) of forkhead box P3 (FOXP3) within Treg (CD4^+^FOXP3^+^CD127^−^) was positively correlated with hospitalization duration and peak WBC count. Notably, FOXP3 MFI of Tregs measured during the convalescent phase at 6 to 12 months after infection remained associated with the hospitalization duration. These findings suggest that an exaggerated Treg response may be associated with the progression toward severe HFRS (66).

Mucosal-associated invariant T (MAIT) cells are an innate-like T cell subset that can undergo changes in both frequency and activation status during hantavirus infection. In patients with HTNV-induced HFRS, peripheral blood CD8^+^ MAIT cells are markedly depleted and activated, and the extent of these changes correlates with disease severity (67). Consistent with these observations, Maleki et al. analyzed a Swedish longitudinal cohort of 24 patients with PUUV-induced HFRS and demonstrated that PUUV infection triggered pronounced activation of circulating MAIT cells, as evidenced by CD69 upregulation and enhanced granzyme B expression. Moreover, the degree of MAIT cell activation was positively correlated with plasma IL-6 levels and inversely correlated with PLT, suggesting that MAIT cells may contribute to the exacerbation of HFRS (68). Additionally, HTNV can directly infect CD8^+^ T cells. In a clinical cohort of 280 patients, Liu et al. found that the number of circulating HTNV-infected CD8^+^ T cells was proportional to disease severity, suggesting that virus-infected CD8^+^ T cells may serve as an auxiliary marker for predicting severe HFRS (69).

Taken together, the evidence for T cell based early warning in HFRS can be organized into two main lines. The first is that a weak antigen-specific T cell response indicates insufficient antiviral clearance, predicting a higher risk of disease progression. The second is that the aberrant expansion or activation of particular T cell subsets, including CD4^+^CD8^+^ T cells, activated Tregs, and virus infected CD8^+^ T cells, directly reflects the depth of immunopathological injury, thereby further augmenting the warning value of peripheral blood T cell profiling.

#### 3.2.2 B-cell kinetics and antibody responses

The rapidity of humoral immune initiation is a key determinant of the clinical outcome of HFRS. Li et al. performed a longitudinal analysis of 23 HTNV-HFRS patients in Baoji and showed that severe patients characteristically exhibited delayed anti-hantavirus nucleocapsid protein (NP)-specific IgG responses, with antibody titers significantly negatively correlated with viral load. Compared to moderate patients, severe patients showed markedly delayed expansion of activated and memory B cells, with plasmablast peak delayed by about two weeks, resulting in insufficient B-cell receptor (BCR) repertoire diversity reconstruction during convalescence (70). In PUUV infection, the insufficient IgG response is also an important marker for severity prediction. Pettersson et al. analyzed 105 PUUV-infected patients in Sweden and found that specific IgG titers at admission showed a strong inverse correlation with disease severity. These findings highlight that a delayed or insufficient early virus-specific IgG response might serve as an important predictor of severe HFRS (71). Recently, the role of mucosal immunity in HFRS pathogenesis is being increasingly recognized. Circulating mucosal-type IgA responses in PUUV infection are positively correlated with AKI severity, implying that mucosal immune activation triggered by respiratory viral entry may extend to the systemic compartment and contribute to renal injury (72).

#### 3.2.3 Monocyte and macrophage activation

The monocyte-macrophage system plays an important role in HFRS immunopathology, and abnormalities in subset distribution and activation status are closely associated with severity. Human peripheral blood monocytes can be divided into three subsets, namely classical (CD14^++^CD16^−^), intermediate (CD14^++^CD16^+^), and non-classical (CD14^+^CD16^++^). In a flow cytometric analysis of 29 patients with HTNV-HFRS in Xi’an, Jia et al. found that the proportions of the three subsets did not change with severe/critical versus mild/moderate stratification. Notably, the MFI of CD31, also called platelet endothelial cell adhesion molecule-1 (PECAM-1), on classical and intermediate monocytes was significantly lower in severe/critical than in mild/moderate patients, whereas CD31 expression on nonclassical monocytes remained unchanged across disease severity (73). In a separate flow cytometric study of 41 HFRS patients, Li et al. found that intermediate monocytes were significantly elevated during the acute phase, with more pronounced expansion in severe/critical patients (13).

Aberrant activation of intermediate monocytes is a core event in HTNV-driven myeloid activation, with changes in CD163 expression being particularly prominent. In the cohort of 41 HFRS patients, Li et al. further found that CD163 expression intensity on intermediate monocytes was significantly higher in severe/critical than in mild/moderate patients, and that the frequency of the CD14^++^CD16^+^CD163^+^ subset was positively correlated with WBC, BUN, and Cr (13). As a product of CD163 shedding, soluble CD163 (sCD163) has also shown remarkable predictive potential in two independent HTNV-HFRS cohorts from Tangdu Hospital in Xi’an. Wang and colleagues studied 81 HFRS patients and found that sCD163 levels during the acute phase increased with disease severity, with severe/critical levels significantly higher than mild/moderate. Notably, 60.8% of severe/critical patients had sCD163 >4 mg/L. sCD163 was positively correlated with WBC, Cr, and BUN, but strongly negatively correlated with PLT. Also, it showed synchronous elevation with IL-6 and IFN-γ (74). In a longitudinal study of 66 HFRS patients, Zhang et al. further demonstrated through dynamic monitoring that sCD163 rose from the febrile phase and peaked during the oliguric phase. The peak level was positively correlated with Cr, BUN, and hospitalization duration, and negatively correlated with minimum PLT. Moreover, this elevation was more pronounced in patients with severe AKI (75). Together, these results indicate that monitoring CD163 changes from membrane phenotype to plasma soluble form is an effective strategy for evaluating the degree of myeloid activation and renal injury risk in HFRS.

The polarization direction and surface molecule changes of monocytes also have predictive significance. Ma et al. reported that M1-like monocytes defined as CD14^+^CD16^+^ were expanded during the acute phase of HTNV infection, and their proportion was positively correlated with disease severity. In severe/critical patients, the intracellular expression of TNF-α/IL-8/IL-10 in M1-like monocytes was enhanced, and the frequency of TNF-α^+^ monocytes correlated with disease severity. These findings suggest that the M1-like inflammatory phenotype may serve as an important driver of the cytokine storm and contribute to progression toward severe outcomes (76). CD14^++^CD16^+^CD206^+^ monocytes were elevated during the acute phase, but their frequency was higher in mild/moderate than in severe/critical patients (13). In a flow cytometric study of 85 patients with HTNV-HFRS conducted in Xi’an, Tang et al. observed a significant expansion of CD226^−^ inflammatory monocytes (CD14^++^CD226^−^) in the peripheral blood during the acute phase. The proportion of this subset was higher in severe/critical patients, positively correlated with plasma HTNV load and WBC, and strongly negatively correlated with PLT. Notably, surface expression of HLA-DR/DP/DQ and CD80 on this subset was markedly lower than that on the CD226^+^ subset, indicating that the loss of CD226, which affects antigen presentation, is closely related to severe progression (77). Collectively, the predictive evidence from the monocyte-macrophage system mainly centers on the aberrant activation of intermediate monocytes, elevated CD163 and sCD163 levels, M1-like polarization, and CD226 loss. Among these indicators, sCD163 is particularly attractive for its detectability in plasma.

#### 3.2.4 Neutrophil activation and degranulation

In recent years, the role of neutrophils in HFRS severity prediction has received increasing attention. The degree of neutrophil activation can be quantified by surface CD64 expression levels, which is clinically interpreted using the neutrophil CD64 index (nCD64). A study evaluated the value of neutrophil nCD64 in predicting severity in 64 HFRS patients and found that nCD64 expression was significantly upregulated in severe patients during the acute phase. The index achieved an AUC of 0.769 for predicting severe HFRS, outperforming traditional markers such as CRP, Cr, and WBC (78). Neutrophil activation is also reflected in degranulation products. Li et al. analyzed 48 HTNV-HFRS patients and showed that plasma myeloperoxidase (MPO) was significantly elevated during the acute phase, with the median in severe/critical patients significantly higher than in mild/moderate patients (443.95 vs. 320.40 ng/mL). MPO was positively correlated with WBC, neutrophil count, BUN, Cr, and UA, and strongly negatively correlated with PLT (79). Although studies specifically focusing on neutrophils remain limited, nCD64 and MPO as activation readouts have demonstrated certain predictive efficacy, and their clinical usefulness needs to be confirmed in larger cohorts.

#### 3.2.5 Complement-activation-related biomarkers

Complement activation has been increasingly recognized as a key immunological event in the pathogenesis of severe HFRS. Two markers are important for assessing this process. SC5b-9 is the soluble form of the membrane attack complex, which is the terminal product of complement activation. C3 is a central component of the complement cascade and its levels drop when the alternative pathway is activated. In the study of Sane et al., plasma SC5b-9 concentrations were significantly elevated in the acute stage of patients with PUUV-HFRS, while acute-stage C3 levels were markedly reduced relative to convalescent values, confirming robust alternative pathway activation during the early phase of PUUV infection. SC5b-9 levels were significantly higher in patients with chest X-ray abnormalities suggestive of pulmonary capillary leakage. Moreover, maximum SC5b-9 correlated positively with hospitalization duration, peak leukocyte count, weight gain reflecting fluid retention, and serum creatinine, and negatively with platelet count. Minimum C3 levels showed a strong inverse correlation with interleukin-6 (80). These findings indicate that complement activation may actively participate in the progression of PUUV-HFRS to critical illness by disrupting endothelial integrity, promoting vascular leakage, and amplifying inflammations.

#### 3.2.6 Other immune effector molecules

Beyond classical cytokines and immune cell subset markers, a variety of other immune effector molecules have also demonstrated significant value in predicting severe outcomes in HFRS. High mobility group box 1 protein (HMGB1) is a late-phase inflammatory mediator that is released during cell necrosis or actively secreted upon inflammatory stimulation. Resman et al. analyzed acute-phase serum from 48 PUUV-HFRS patients and found that the median HMGB1 level in the severe group reached 138 ng/mL, significantly higher than 7.3 ng/mL in the mild group, suggesting that HMGB1 has potential severity stratification value in PUUV-HFRS (81). Adenosine deaminase (ADA), a key enzyme in purine metabolism, can regulate T cell activation through adenosine receptor pathways. Zhu et al. retrospectively analyzed 124 HTNV-HFRS and reported that ADA levels decreased with disease remission. Notably, the two patients who died had elevated ADA levels on admission, with values of 28 U/L and 52 U/L respectively, and in one of them, the level rose further to 58 U/L during disease progression, suggesting that persistently high ADA may reflect uncontrolled disease activity (82). Additionally, Liu et al. analyzed 99 HTNV-HFRS patients at Tangdu Hospital in Xi’an found that plasma levels of soluble CD100 (sCD100) during the acute phase increased in a stepwise manner according to the clinical classification of HFRS. sCD100 was strongly negatively correlated with platelet count and significantly positively correlated with WBC, creatinine, and BUN, suggesting sCD100 as a candidate warning marker for HTNV-HFRS disease severity (83).

In summary, immunological biomarkers have provided broad and consistent evidence for the early prediction of severe HFRS. At the cytokine level, the sustained elevation of proinflammatory factors (IL-6, TNF-α) and the decrease of protective factors (IFN-γ, TGF-β) together constitute the core immunological signature of severity. At the immune cell level, attenuated antigen-specific T cell responses indicate insufficient antiviral clearance, while aberrant expansion of CD4^+^CD8^+^ T cells, activated Tregs, and virus infected CD8^+^ T cells directly reflects the depth of immunopathological injury, thereby further augmenting the warning value of T cell profiling. Regarding B cell compartment, delayed NP-specific IgG responses reveal the association between insufficient humoral clearance and severity. In the monocyte-macrophage system, aberrant activation of intermediate monocytes and elevated sCD163 levels are closely associated with severe disease, underscoring their value as predictive indicators of critical HFRS. Neutrophil activation markers, including nCD64 and MPO, have demonstrated predictive value for severity. Complement activation, as reflected by elevated SC5b-9 and reduced C3, may further contribute to the immunopathological signature of severe HFRS. Furthermore, other immune effector molecules such as HMGB1, ADA, and sCD100 have been shown to correlate with disease severity and may serve as complementary warning indicators. Notably, viral load during the febrile phase was significantly correlated with disease severity (84). These findings suggest that combining viral load with immune activation markers may enable a more comprehensive assessment of the risk of severe HFRS. However, most of these biomarkers depend on specialized detection platforms such as flow cytometry, enzyme-linked immunosorbent assay (ELISA), or mass spectrometry, which are not yet routinely available at all hospital levels, and their clinical translation depends on the wider dissemination and standardization of these assays in clinical practice.

## 4 Emerging biomarkers

### 4.1 Emerging markers of endothelial barrier disruption

Endothelial cell activation and injury represent core processes in the pathophysiology of HFRS, and related emerging markers reflect the degree of vascular barrier disruption from different perspectives.

Soluble vascular cell adhesion molecule-1 (sVCAM-1) is a classical marker of endothelial activation. In a study of 52 HTNV-HFRS patients, Qi et al. discovered that plasma sVCAM-1 levels had a positive correlation with both disease severity and renal injury markers (BUN, Cr), suggesting its potential as a warning indicator for HFRS severity (85). Soluble vascular endothelial growth factor receptor 2 (sVEGFR-2) acts as a decoy receptor for vascular endothelial growth factor (VEGF) in plasma, sequestering free VEGF and thereby limiting vascular leakage. Pal et al. analyzed 58 PUUV-HFRS patients and found that initial plasma sVEGFR-2 levels were significantly lower in patients with severe disease than in those with mild disease. Given that plasma sVEGFR-2 is susceptible to systemic endothelial activation and platelet metabolism, the researchers further measured urinary sVEGFR-2 and observed significantly higher levels in the severe group, proposing urinary sVEGFR-2 as a candidate marker for predicting severe HFRS (86). Vascular endothelial cadherin (VE-cadherin) is a core protein of endothelial intercellular adherens junctions, and its serum level can reflect endothelial barrier integrity. In a study of PUUV-HFRS, it was found that serum VE-cadherin levels decreased with the severity of the disease and were negatively correlated with circulating endothelial cell counts, suggesting exacerbated endothelial desquamation. Additionally, a T to C missense mutation at position c.1550 of the VE-cadherin gene was found to be associated with HFRS severity (87).

Angiopoietins are widely recognized as critical regulators of both angiogenesis and the preservation of vascular endothelial integrity. Ang-1 serves to reinforce intercellular junctions and maintain vascular stability, whereas Ang-2 exerts an opposing influence by antagonizing Ang-1, thereby promoting junctional disassembly and compromising the integrity of the endothelial monolayer (88, 89). Therefore, an elevated Ang-2/Ang-1 ratio may reflect endothelial barrier damage and an enhanced tendency of vascular leakage. In a PUUV infection cohort, the Ang-2/Ang-1 ratio was positively correlated with serum creatinine and negatively correlated with serum albumin and platelet (90). This indicates that Ang-2/Ang-1 imbalance may participate in the pathological progression of hantavirus disease to severe forms by disrupting the endothelial barrier and increasing capillary permeability.

Dentin matrix protein 1 (DMP1) can degrade basement membrane components and intercellular endothelial junctions through matrix metalloproteinase-mediated mechanisms, and its elevation may directly contribute to increased vascular permeability. Shi et al. demonstrated that in an HTNV-HFRS cohort, serum DMP1 peaked during the oliguric phase and was highest in the critical severity group. The overall serum DMP1 levels also showed significant positive correlations with BUN, Cr, CysC, and VEGF, supporting its potential utility as a candidate biomarker for HTNV-HFRS severity (91).

In summary, these endothelial injury markers reflect the disruption of the vascular barrier in HFRS from multiple complementary perspectives, including endothelial activation indicated by sVCAM-1, perturbation of the VEGF signaling pathway captured by sVEGFR-2, impairment of interendothelial junctions reflected by VE-cadherin, endothelial barrier damage and vascular leakage tendency indicated by the Ang-2/Ang-1 ratio, and degradation of the basement membrane represented by DMP1. Their significant correlations with disease severity and renal dysfunction support their potential as early warning biomarkers.

### 4.2 Emerging markers of acute kidney injury

Cr, as a traditional diagnostic marker for AKI, is inherently delayed in detecting early renal damage and usually rises only after the GFR has dropped by about 50% (92). Therefore, identifying novel biomarkers that can reflect renal injury earlier and more sensitively is important for HFRS severity prediction.

CysC is superior to Cr in detecting early decline of glomerular filtration function. Ma et al. detected 32 HTNV-HFRS patients and found that serum CysC levels were already significantly elevated during the febrile/hypotensive phase while Cr had not yet risen. During the acute phase, urinary CysC elevation exceeded that of Cr, with the most pronounced elevation observed in patients with severe or critical disease. Urinary CysC levels in the acute phase were also positively correlated with the severity of subsequent renal impairment (93). A prospective study of 44 PUUV-infected patients in Sweden further confirmed that plasma CysC and urinary alpha-1-microglobulin could effectively predict severe AKI onset during early disease, with individual AUCs of 0.72 and 0.73, respectively, both of which outperformed plasma Cr (AUC 0.65) and urinary albumin (AUC 0.53) measured during the same period (94).

Regarding renal tubular injury, Bunz et al. employed a cohort of 61 PUUV-associated NE patients in Germany and found that urinary neutrophil gelatinase-associated lipocalin (uNGAL) is a sensitive indicator for predicting AKI severity. The admission uNGAL predicted severe AKI with an AUC of 0.81 and showed a positive correlation with proteinuria levels (95). Indoleamine 2,3-dioxygenase (IDO), a rate-limiting enzyme in tryptophan metabolism, has been reported to promote the apoptosis of renal tubular epithelial cells, and its activity is thought to indicate the severity of renal injury (96). Outinen et al. confirmed that PUUV-induced IDO overactivation (serum kynurenine/tryptophan ratio >202) was significantly correlated with disease severity, predicting severe renal failure with an AUC of 0.84. Patients with high enzyme activity also experienced prolonged hospitalization and a significantly increased risk of dialysis (97).

### 4.3 Emerging markers of metabolic, oxidative, and systemic injury

Beyond endothelial and renal injury markers, novel markers spanning metabolism, oxidative stress, glycocalyx integrity, and neuroendocrine regulation have also shown promise in the early prediction of severe HFRS.

Adiponectin (APN) is an anti-inflammatory metabolic hormone secreted by adipocytes. Du et al. analyzed 105 patients with HTNV-HFRS in Xi’an and found that plasma APN levels during the acute phase were negatively correlated with disease severity. Plasma APN levels decreased progressively with increasing disease severity. Specifically, levels dropped from approximately 12.0 ng/mL in mild/moderate patients to 7.5 ng/mL in the severe group. APN was also negatively correlated with WBC and hematocrit (HCT), and positively correlated with PLT, ALB, and UA, confirming its potential value as a marker linking metabolic and inflammatory changes to HFRS severity (98). Mantula et al. analyzed 79 PUUV-infected patients in Finland and demonstrated that plasma resistin, a macrophage-derived inflammatory adipokine, rose with proteinuria levels, yielding an AUC of 0.82 for predicting severe AKI (99). YKL-40 is an inflammatory glycoprotein secreted by activated macrophages, neutrophils, and vascular smooth muscle cells. Outinen et al. reported that YKL-40 levels were significantly elevated during the acute phase of PUUV infection, with peak values significantly positively correlated with peak Cr, IL-6, CRP, and hospitalization duration. These findings suggest that YKL-40 may serve as a serum marker reflecting acute-phase inflammatory burden and predicting AKI severity (100).

Regarding oxidative stress, superoxide dismutase (SOD) is an important enzyme in the antioxidant defense system. Tian et al. retrospectively analyzed 149 HTNV-HFRS patients and 30 healthy controls and found that SOD levels were significantly lower in severe/critical patients than in mild/moderate patients. Further survival analysis showed that patients with admission SOD below 88.6 U/mL had significantly elevated mortality risk, with SOD predicting death at an AUC of 0.8178. Multivariate analysis also identified SOD as an independent risk factor for HFRS mortality (101). Together, these findings highlight the potential of SOD as a clinically accessible biomarker for early risk stratification and prognostication in HFRS.

The importance of glucose homeostasis disruption in HFRS is also gaining attention. Shi et al. retrospectively analyzed 684 HTNV-HFRS patients in Baoji and found that both serum glucose levels and urine glucose test strip positivity increased progressively with the four clinical severity grades (102). Tietavainen et al. studied 185 PUUV-HFRS patients in Finland and stratified them according to peak plasma glucose levels below or above 7.8 mmol/L. They found that the hyperglycemia group had lower PLT, higher peak Cr levels, a greater frequency of shock and pulmonary infiltration, and longer hospitalization. In multivariable analysis, peak blood glucose was independently associated with capillary leak markers, thrombocytopenia, and inflammatory indicators. These findings suggest that bedside glucose as an accessible but easily overlooked metabolic signal deserves reevaluation in early severity identification (103).

Glycocalyx disruption is a key pathological process driving capillary leakage. Soluble syndecan-1 (sCD138) has been established as a specific marker of glycocalyx injury, and its plasma levels have been shown to correlate significantly with HFRS severity (104). Cell-free DNA (cfDNA) refers to DNA fragments released into the circulation following apoptosis or necrosis and can non-specifically reflect the overall burden of tissue injury. Yi et al. analyzed 76 HTNV-HFRS patients in Xi’an and showed that plasma cfDNA levels were significantly elevated in severe/critical patients during the febrile/hypotensive phase, positively correlated with HTNV viral load, peak WBC, and Cr, and negatively correlated with minimum PLT (105).

Some systems that have historically been overlooked are now proving valuable in the early warning of severe HFRS. At the central nervous system level, Hautala et al. found that elevated cerebrospinal fluid neopterin in PUUV-HFRS correlates positively with Cr and hospital stay, and negatively with PLT (106). A cohort study by Tarvainen et al. further showed that the acute phase of PUUV infection is frequently accompanied by complex neuroendocrine disturbances, including both central and peripheral gonadal and thyroid dysfunction. More importantly, these hormonal deficiencies were strongly linked to indicators of coagulation and fibrinolysis imbalance, such as increased D-dimer levels and heightened platelet consumption, indicating that disruption of endocrine homeostasis is also a crucial factor driving disease progression toward severe forms (107).

Taken together, these emerging indicators extend the early warning system for severe HFRS by capturing multiple pathophysiological dimensions, including metabolic imbalance, oxidative stress, glycocalyx degradation, cell injury, and neuroendocrine disturbance.

## 5 Conclusion and discussion

HFRS severity progression is characterized by acute onset and rapid deterioration, and given the lack of specific antiviral therapy currently available, early identification of high-risk patients is critical for improving prognosis. This review addresses this core clinical challenge by comprehensively synthesizing predictive evidence from three dimensions, namely routine clinical indicators, immune dysregulation-related markers, and emerging biomarkers. It delineates the knowledge landscape of HFRS severity prediction.

Bedside indicators and routine clinical parameters offer the greatest accessibility and constitute the foundation of the warning system. Expert Consensus has identified high fever, severe gastrointestinal symptoms, altered mental status, conjunctival chemosis, and obvious bleeding tendency as severity warning indicators (10). Pleural effusion and qSOFA score also provide independent warning information (19). Among laboratory indicators, thrombocytopenia and leukocytosis represent the most classic combination for early warning (11, 22). PCT demonstrates robust predictive value for severity and mortality (27), while CRP has limited independent predictive value (28). Elevated D-dimer and sTM reflect early hyperfibrinolysis and endothelial injury (23, 31). APRI and ferritin add warning information from the perspectives of hepatic injury and inflammatory burden (7, 40). Building on these indicators, multi-marker prediction models have been shown to substantially improve discriminative performance, highlighting the integration of multidimensional indicators as an effective strategy for enhancing the early warning capacity for severe HFRS (39, 41–43).

Immunological markers also play a critical role in HFRS severity prediction. The elevation of IL-6 and TNF-α combined with the decrease of IFN-γ and TGF-β constitutes the core cytokine imbalance signature (49, 50, 52–54). Aberrant upregulation of MCP-1 and CXCL10 reflects excessive innate immune activation (56, 57). At the T cell level, attenuated antigen-specific responses and abnormal expansion of CD4^+^CD8^+^ T cells provide warning signals of severe HFRS (64). Delayed IgG responses are also a characteristic feature of severity (71). sCD163, as a monocyte-macrophage activation marker, is positively correlated with renal injury and negatively correlated with PLT, suggesting its potential in the prediction of severe HFRS (74).

Emerging biomarkers extend the early warning capacity by encompassing pathophysiological dimensions that conventional measures do not completely cover, including endothelial injury, renal tubular injury, oxidative stress, and metabolic dysregulation. Severe AKI can be predicted by CysC and uNGAL prior to a noticeable elevation in Cr (93–95). sVCAM-1 and sCD138 can reflect the degree of vascular barrier injury, hinting at the potential for severe HFRS (85, 104). SOD and other oxidative stress markers, along with APN and resistin, provide additional warning signals from the perspectives of antioxidant defense and lipid metabolism disruption (98, 99, 101).

Each category of biomarkers has its own strengths and limitations. Bedside signs and routine clinical parameters offer strong accessibility and reproducibility, but they often show abnormalities only when organ damage is already present. Immunological markers can provide earlier insight into severe warning, but their measurement often relies on specialized detection platforms such as flow cytometry and ELISPOT. Emerging biomarkers expand the scope of early warning, however, most of them remain at the single-center exploratory stage, and their independent predictive value and clinical feasibility still need further validation.

Despite the relatively abundant evidence summarized above, the current literature on severe HFRS prediction still has several limitations. First, heterogeneity in study design remains the most critical barrier to the clinical translation of biomarkers in severe HFRS prediction. Most existing studies on early warning of severe HFRS consist of single-center retrospective cohort studies with small sample sizes, and there has been significant variation in inclusion and exclusion criteria, timing of biomarker measurements, and data collection methods. Although this design is reasonable under resource constrained conditions, its limitations are equally evident. Single-center data are deeply linked to the patient composition, clinical protocols, and laboratory capacity of the local hospital, so cutoffs derived in one institution may not be transferable to other regions. Single-center designs also make it difficult to effectively control the timing of sample collection. Given the distinct clinical phases of HFRS, the significance of a given biomarker may differ substantially between different stages. Furthermore, small sample sizes tend to introduce optimistic bias during the screening of candidate markers, and internal validation by itself cannot reliably indicate how a predictive model will perform in a different population (108, 109).

Second, the existing research provides insufficient coverage of special populations. HFRS can occur at any age, yet the majority of current studies have focused on adults, particularly young and middle-aged individuals. However, the elderly, children, pregnant women, and patients with underlying comorbidities constitute a substantial proportion of the affected population, and their clinical phenotypes, rates of disease progression, and outcomes differ markedly from those of the general adult patient. For example, older patients often have diminished renal functional reserve and a high prevalence of hypertension and atherosclerosis. Even when viral load and inflammatory intensity are not particularly high, they are more likely to progress rapidly to AKI and heart failure (110, 111). Children represent the opposite scenario. Their renal tubular compensatory capacity is strong, and although fever and gastrointestinal symptoms tend to be more prominent than in adults, the incidence and severity of AKI are generally lower (112). Pregnant women add further complexity, as placental hemodynamic changes and fetal considerations place their circulatory volume, coagulation system, and immune status in a unique and dynamically balanced state (113, 114). Given these marked differences, establishing dedicated subgroup cohorts for special populations and developing stratified or subgroup-specific early warning tools represents an important direction for improving the applicability of predictive models in clinical practice.

Lastly, the limited accessibility of detection platforms and the lack of evidence for incremental value remain the principal barriers to clinical translation. Many promising markers, such as CD4^+^CD8^+^ T cells, nCD64, cfDNA and soluble syndecan-1, often depend on flow cytometry or other high-throughput platforms. These techniques are time-consuming and costly, making their routine use difficult in primary care settings. More importantly, most existing studies have only examined associations between these markers and clinical outcomes, and whether adding them to conventional indicators truly improves the warning capacity remains unclear. Future work should promote point-of-care testing by integrating markers such as soluble CD163, cystatin C, nCD64, and procalcitonin into microfluidic, chemiluminescence, or immunochromatographic devices, shifting detection from central laboratories to the bedside. A tiered warning pathway should also be established, in which primary care institutions use routine indicators for initial screening, regional hospitals incorporate moderately accessible biomarkers for risk stratification, and large tertiary hospitals integrate immune and endothelial markers for refined management. Within this framework, prospective interventional studies are needed to determine whether biomarker-guided decisions can improve the accuracy of early warning of severe HFRS. Such evidence would clarify the incremental value of these markers and provide a solid foundation for their adoption in clinical practice.
