# Supplementary material for: Early warning indicators for severe hemorrhagic fever with renal syndrome
Source: Front Immunol. 2026 Oct 26;17:1986264. doi: 10.3389/fimmu.2026.1986264 (PMC13746193; doi:10.3389/fimmu.2026.1986264)
Supplement: Supplementary file 1 [file Table1.docx]

Table S1. Comparison of published predictive models for predicting severity or prognosis in HFRS

| **Study** | **Virus** | **Cohort, n** | **Model components / predictors** | **Outcome** | **AUC and validation** |
| --- | --- | --- | --- | --- | --- |
| Kim et al., 2007 [41] | HTNV | 61 (oliguric ARF 24 and non-oliguric ARF 37) | WBC ≥14×10^9^/L; AST ≥110 U/L; microscopic hematuria ≥5/HPF | Oliguric ARF (urine output <400 mL/24 h) | / |
| Du et al., 2014 [42] | HTNV and SEOV | 356 (critical 75; severe 85; moderate 126; mild 70) | WBC; AST; PT; Fib | Death vs survival | Combined AUC 0.945 |
| Latus et al., 2015 [43] | PUUV | 456 (impaired kidney function 319; normal kidney function 137) | Thrombocytopenia <90×10^9^/L (2 points); 12-fold CRP increase (1 point); proteinuria, ACR >0.25 g/g Cr (1 point) | Severe AKI (RIFLE Injury or Failure) in patients with normal kidney function at diagnosis | AUC 0.71; internal 5-fold cross-validation AUC 0.67 |
| Duan et al., 2024 [27] | / | 164 (severe 50; mild 114) | WBC; PLT; PCT | Severe + critical vs mild + moderate HFRS | Combined AUC 0.900 |
| Wang et al., 2026 [18] | HTNV | 570 (training 344: critical 45, non‑critical 299; validation 226; random split) | Hypotensive shock; myoglobin; neutrophils | Critical vs noncritical HFRS | Nomogram: 0.889 / 0.911; scoring scale: 0.861 / 0.906, training/validation |
| Yu et al., 2017 [29] | / | 384 (severe 50; mild 334) | Four-factor combination: PLT, PCT, TB, FOBT; H-SOFA: SOFA plus graded FOBT | Severe (serious + critical) vs mild (mild + moderate) HFRS | Four-factor AUC 0.890; SAPS II 0.900; SOFA 0.960; H-SOFA 0.980 |
| Yang et al., 2021 [12] | / | 155 (derivation 109: severe 63, mild 46; temporal validation 46: severe 23, mild 23) | Neutrophils; Hb; PLT; Cr; Ca; dyspnea | Severe + critical vs mild + moderate HFRS | Derivation 0.969; validation 0.934 |
| Xie et al., 2022 [44] | / | 101 (severe 38; mild 63) | WBC; ALB; PT; CK-MB | Severe + critical vs mild + moderate HFRS | Combined AUC 0.950 |
| Chen et al., 2023 [45] | / | 235 (severe 131; mild 104); random 80% model-development / 20% validation split | Febrile: eGFR (positive), urine WBC (maximum), Na (mean), urine conductivity (mean), urine epithelial cells (fluctuation), urinary casts (maximum). Nonfebrile: eGFR (positive), Na (mean), urine conductivity (mean), cystatin C (maximum), myocardial enzymes/CK (maximum) | Severe vs mild HFRS | Febrile: 0.92 / 0.96; nonfebrile: 0.93 / 0.98 (reported as internal / external validation AUCs) |
| Ma et al., 2025 [19] | / | 551 (training 385; validation 166; random 7:3 split) | RBC ≥4.87×10^12^/L; age ≥61 y; two-stage overlap; qSOFA ≥2; AST ≥161 IU/L; three-stage overlap | 30-day mortality | Training 0.9249; validation 0.9102; bootstrap internal AUC 0.886 |
| Dou et al., 2025 [33] | HTNV | 342 (derivation 259: noncritical 210, critical 49; externally validated 83) | WBC, LYM, ALB, CK, Cr, TEG K, TEG alpha-angle | Critical vs noncritical HFRS | Derivation AUC 0.9058; bootstrap internal C-index 0.866; externally validated AUC 0.762 |
| Huang et al., 2025 [14] | / | 191 (severe 42; mild 149) | PCT; ALB; platelet-to-albumin ratio (PAR); pleural effusion | Severe vs mild HFRS | AUC 0.890 |
| Huang et al., 2025 [46] | / | 373 (survival 341; death 32) | PT; FT3; LAR; WBC | 30-day mortality | C-index/AUC 0.946 |
| Zhao et al., 2025 [39] | HTNV | 176 (critical 40; severe 48; moderate 43; mild 45) | BNP; PLT; urea; Cr | Serious (severe + critical) vs non-serious (mild + moderate) HFRS | BNP alone 0.853; four-index combination 0.921 |

Abbreviations: ACR, albumin-to-Cr ratio; AKI, acute kidney injury; ALB, albumin; ARF, acute renal failure; AST, aspartate aminotransferase; AUC, area under the receiver operating characteristic curve; BNP, B-type natriuretic peptide; Ca, calcium; CK, creatine kinase; CK-MB, creatine kinase-MB; CRP, C-reactive protein; eGFR, estimated glomerular filtration rate; Fib, fibrinogen; FOBT, fecal occult blood test; FT3, free triiodothyronine; Hb, hemoglobin; LAR, lactate dehydrogenase-to-albumin ratio; LYM, lymphocyte count; PAR, platelet-to-albumin ratio; PCT, procalcitonin; PLT, platelet count; PT, prothrombin time; qSOFA, quick Sequential Organ Failure Assessment; RBC, red blood cell count; Cr, creatinine; SOFA, Sequential Organ Failure Assessment; TB, total bilirubin; TEG, thromboelastography; WBC, white blood cell count.
